# Tuberculosis in Swiss captive Asian elephants: microevolution of *Mycobacterium tuberculosis* characterized by multilocus variable-number tandem-repeat analysis and whole-genome sequencing

**DOI:** 10.1038/s41598-017-15278-9

**Published:** 2017-11-07

**Authors:** Giovanni Ghielmetti, Mireia Coscolla, Maja Ruetten, Ute Friedel, Chloé Loiseau, Julia Feldmann, Hanspeter W. Steinmetz, David Stucki, Sebastien Gagneux

**Affiliations:** 1 Institute of Veterinary Bacteriology, University of Zurich, CH-8057, Zurich, Switzerland; 2 Swiss Tropical and Public Health Institute, CH-4002, Basel, Switzerland; 3 University of Basel, CH-4002, Basel, Switzerland; 4 Institute of Veterinary Pathology, University of Zurich, CH-8057, Zurich, Switzerland; 5 Present Address: PathoVet AG, CH-8317, Tagelswangen, Switzerland

## Abstract

Zoonotic tuberculosis is a risk for human health, especially when animals are in close contact with humans. *Mycobacterium tuberculosis* was cultured from several organs, including lung tissue and gastric mucosa, of three captive elephants euthanized in a Swiss zoo. The elephants presented weight loss, weakness and exercise intolerance. Molecular characterization of the *M. tuberculosis* isolates by spoligotyping revealed an identical profile, suggesting a single source of infection. Multilocus variable-number of tandem-repeat analysis (MLVA) elucidated two divergent populations of bacteria and mixed infection in one elephant, suggesting either different transmission chains or prolonged infection over time. A total of eight *M. tuberculosis* isolates were subjected to whole-genome sequence (WGS) analysis, confirming a single source of infection and indicating the route of transmission between the three animals. Our findings also show that the methods currently used for epidemiological investigations of *M. tuberculosis* infections should be carefully applied on isolates from elephants. Moreover the importance of multiple sampling and analysis of within-host mycobacterial clonal populations for investigations of transmission is demonstrated.

## Introduction

Infections with several members of the *Mycobacterium tuberculosis* complex (MTBC) can cause tuberculosis (TB) in both captive and free-ranging wildlife species, thus representing a considerable zoonotic risk^1–4^. In recent years, because of the broad host range of some MTBC members, numerous reports of MTBC infections in different species, including endangered animals, have been published^5,6^. Modern phylogenetic analysis defined MTBC into seven major lineages adapted to humans and two lineages adapted to various wild and domestic animal species^7^. In contrast to the human-adapted lineages, some animal-adapted members of the MTBC can establish independent transmission chains within different animal species^8^. Some wildlife species are recognized for their role of maintenance host, acting as a reservoir and transmitting the pathogens into domestic livestock, companion animals or human beings^5,9^. Particular attention has been given to badgers or wild boar as reservoirs of *M. bovis*, to red deer in the case of *M. caprae* and wild rodents regarding *M. microti*. The causative agent of human TB, *M. tuberculosis*, is an obligate pathogen known to maintain its life cycle of disease and transmission primarily in human populations^8^. However, infections of *M. tuberculosis* in a variety of domestic animals such as dogs, cattle, horses and parrots^10–13^ as well as in wild animal species such as elephants, rhinoceros, chimpanzee, lesser kudu, tapir and others have been described^1,14–16^. While in some species, such as cattle, *M. tuberculosis* infections are self-limiting and persistence of the pathogen into the population does not occur without exposure to humans^11,17^, cases where *M. tuberculosis* was maintained for a prolonged time within certain wildlife populations have been reported^14^. However, the dynamics of *M. tuberculosis* transmission among wild animals remain unclear.

## Outbreak

Three captive Asian elephants (*Elephas maximus*) kept in a Swiss zoo presented unspecific clinical signs such as weight loss, weakness and exercise intolerance. The elephants were wild-born in Indonesia in the early 1960s, and they were moved to Europe at the age of three, four and eight. Living in a well frequented zoo, high contact with visitors exposed the animals to human pathogens over years. Infection with a member of the MTBC was suggested in February 2015 after clinical examination and a positive screen with DPP® VetTB Assay (Chembio Diagnostic Systems, New York, USA)^18^. This test consists of a lateral flow-based technology that detects serum antibodies specific for antigens MPB83 and ESAT-6/CFP-10 recombinant^19^. All three elephants resulted positive to the test for both antigens and were euthanized because of their deteriorated general condition between June-July 2015 by the official veterinarian attending the zoo animals. Necropsy and analysis of the specimens was carried out within a diagnostic context, meaning that no animals were killed for the purposes of this research project and ethical approval was not necessary. The privacy rights of the zoo were fully protected and the obtained data were de-identified.

The purpose of the present study was to determine whether the three elephants were infected by a single strain of *M. tuberculosis* or by multiple ones, identifying the potential index case and assessing the transmission route between the elephants. Additionally, this study aimed to describe the dissemination and microevolution of the bacterium within the elephant’s body.

## Findings

### Bacterial isolation from trunk wash

Serial trunk wash and subsequent culturing of the samples obtained was performed monthly for the three months prior to euthanasia for each elephant. Direct MTBC RT-PCR (described below) always resulted negative and growth of MTBC members was not detected. *Mycobacterium vaccae* and *Rhodococcus equi* were isolated in two samples at different periods from both Elephant 2 and Elephant 3. Additionally *M. nonchromogenicum* and *M. monacense* were isolated from Elephant 2 and Elephant 3, respectively.

### Necropsy and *M. tuberculosis* isolation

**Figure 1:**
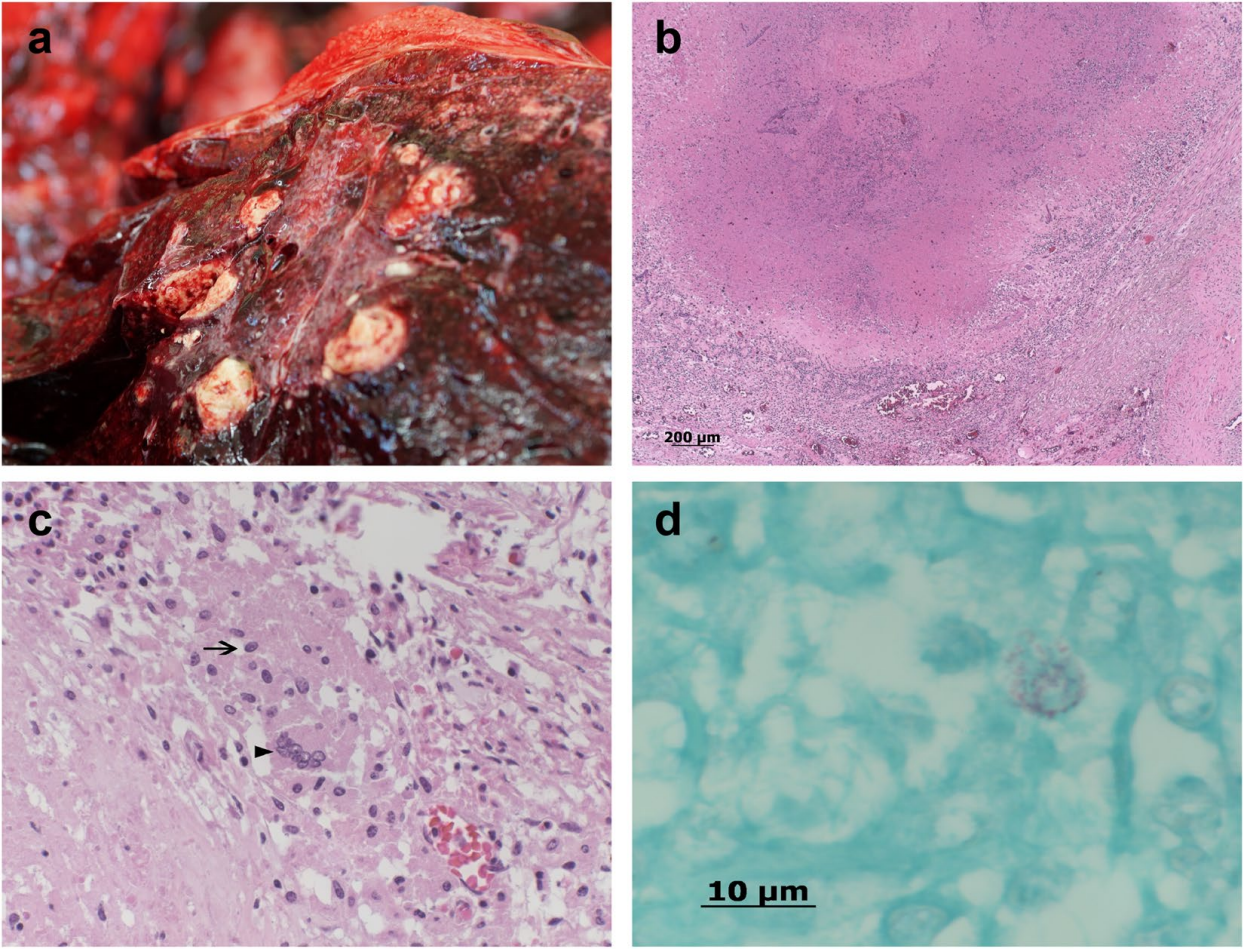
(**a**) Lung, Asian elephant tuberculosis. Multifocal to coalescing yellow-grey nodules, surrounded by connective tissue and a central caseous necrosis were observed during necropsy. (**b**) Histopathological examination revealed organized granulomatous inflammation with caseous necrosis, consistent with chronic TB-lesion, hematoxylin and eosin (HE). (**c**) Inflammation is characterized by numerous epithelioid macrophages (arrow) and several multinucleated Langhans giant cells (arrowhead), HE. (**d**) Alveolar macrophage with intracellular acid fast rods, Ziehl-Neelsen.

All three animals presented multifocal to coalescing yellow-grey necroses of 5 mm to 2 cm in diameter in the lungs (Fig. 1, panel a). The necroses were often mineralized and larger abscesses were encapsulated by fibrosis. All animals showed moderate to severe arthrosis and were mildly anemic. Elephant 2 had severe suppurative discharge from the vagina that was caused by an endometrial cystic hypertrophy with secondary severe pyometra caused by *Streptococcus agalactiae*. The uterus of Elephant 3 showed histopathological features of an adenocarcinoma and presented metastases in the uterine ligaments (Table 1).

**Table 1:** Pathological findings observed in the three Asian elephants.

Elephant 1	Elephant 2	Elephant 3
Multifocal granulomatous pneumonia with abscesses formation		
Extensive proliferative and erosive arthrosis with complete eburnation of hip, knee and carpal joints		
Severe interstitial nephritis with fibrosis	Cystic endometrial hyperplasia	Severe interstitial nephritis with fibrosis and moderate glomerular nephritis
Uremic pneumonia and gastritis	Severe pyometra	Uterine adenocarcinoma with metastases in the uterine ligaments

In addition to pulmonary TB, the three elephants presented various pathological lesions, mostly involving the reproductive system and the locomotor activity.

The lesions of the lungs revealed granuloma formations, displaying round lesions with central coagulative necrosis, surrounded by numerous epithelioid macrophages and several multinucleated Langhans giant cells. The giant cells showed a semilunar organization of their nuclei. Further, in the periphery of the granulomas, lymphocytes, plasma cells and proliferation of fibroblasts were conglomerated (Fig. 1, panels b-c). These findings were consistent with chronic TB lesions, indicating that the three elephants were infected over a long period of time. The lymph nodes were atrophied with a regular architecture but no granulomatous alterations were seen. Sinus histiocytosis was observed in the lymph nodes of the lungs. Acid fast bacilli were only found in alveolar macrophages and in histiocytes of the lung lymph nodes and quantified between scanty and 1+ according to the IUATLD and WHO grading scales (Fig. 1, panel d)^20^.

### Spoligotyping and multilocus variable-number of tandem-repeat analysis (MLVA)

A total of eight isolates cultured from different organs (Table 2) were analyzed by spoligotyping and MLVA. Spoligotyping revealed an identical profile for all the isolates, namely SIT276. SIT276 is characterized by the lack of spacers 25–30 and 33–36. MLVA showed two distinct genotypes and a mixed infection in the right lung of Elephant 3. Locus variations were observed in VNTR577 and VNTR4156 by comparing the isolates from Elephant 1 with those generated from Elephant 2 and 3, respectively (Table 2).

**Table 2:** Allele profiles of the eight isolates cultured from different organs and compared with the reference strain *M. tuberculosis* H37Rv in the 24 MIRU-VNTR standard panel (VNTR 154, 424, 577, 580, 802, 960, 1644, 1955, 2059, 2163b, 2165, 2347, 2401, 2461, 2531, 2687, 2996, 3007, 3171, 3192, 3690, 4052, 4156, 4348) and the additional hypervariable loci VNTR2163a and VNTR3232.

***M. tuberculosis*** **H37Rv**		2	2	4	3′	1	3	2	2	2	5	3	4	2	3	6	1	3	3	3	3	5	5	2	2	2	3
**Elephant 1**	*Left lung*	2	2	**4**	2	4	3	3	2	2	4	3	4	2	2	5	1	4	3	3	3	3	7	**3**	2	2	2
	*Right lung*	2	2	**4**	2	4	3	3	2	2	4	3	4	2	2	5	1	4	3	3	3	3	7	**3**	2	2	2
	*Pharyngeal-swab*	2	2	**4**	2	4	3	3	2	2	4	3	4	2	2	5	1	4	3	3	3	3	7	**3**	2	2	2
**Elephant 2**	*Left lung*	2	2	**5**	2	4	3	3	2	2	4	3	4	2	2	5	1	4	3	3	3	3	7	**2**	2	2	2
**Elephant 3**	*Right lung*	2	2	**4/5**	2	4	3	3	2	2	4	3	4	2	2	5	1	4	3	3	3	3	7	**2/3**	2	2	2
	*Tracheobr.-secretions*	2	2	**5**	2	4	3	3	2	2	4	3	4	2	2	5	1	4	3	3	3	3	7	**2**	2	2	2
	*Gastric mucosa*	2	2	**5**	2	4	3	3	2	2	4	3	4	2	2	5	1	4	3	3	3	3	7	**2**	2	2	2
	*Trunk mucosa*	2	2	**5**	2	4	3	3	2	2	4	3	4	2	2	5	1	4	3	3	3	3	7	**2**	2	2	2

MLVA revealed a double-locus variation in VNTR577 and VNTR4156 by comparing the isolates from Elephant 1 with those generated from Elephant 2 and 3 respectively (bold). Additionally the isolate from the right lung of Elephant 3 showed both mycobacterial populations.

### Whole-genome sequencing

**Figure 2:**
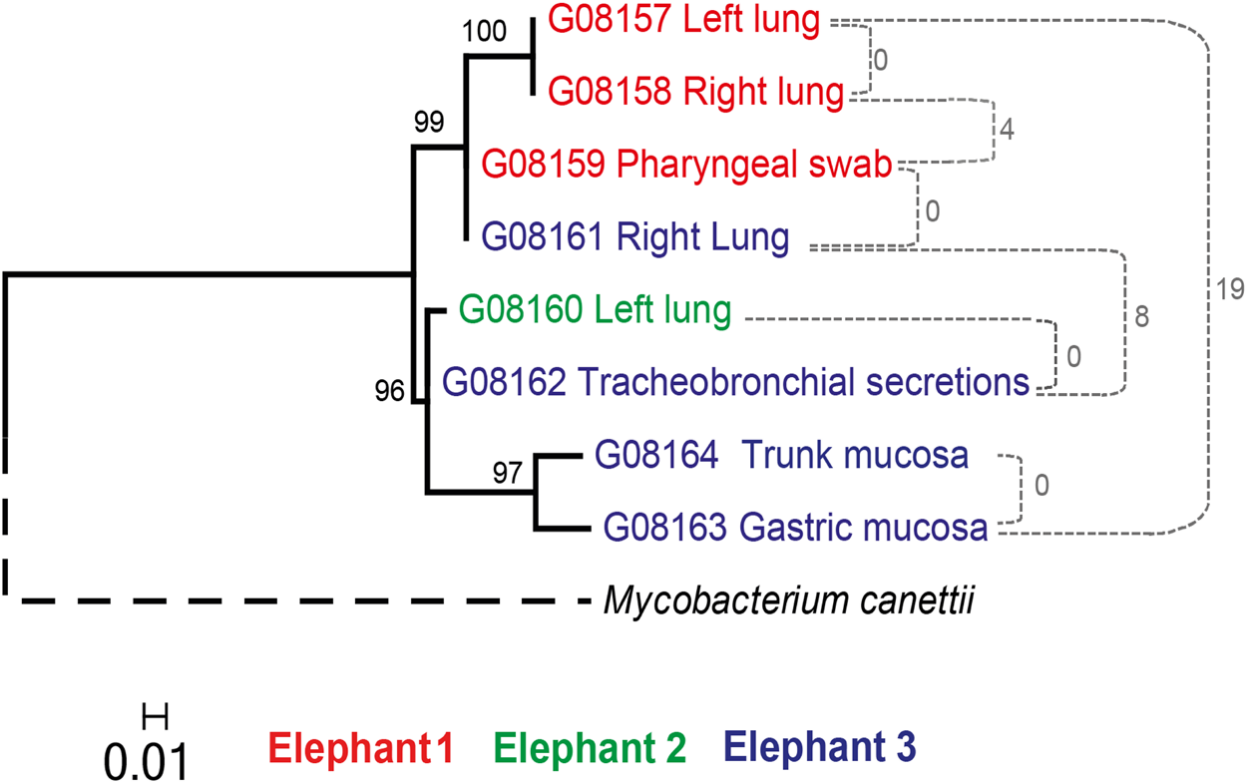
Maximum likelihood phylogeny using the GTR model of evolution obtained with RaxML using a 146 variable positions alignment. *M. canettii* was used as an outgroup and to root the phylogeny. Nodes supports are indicated by 1,000 bootstrap replicates. The maximum number of SNP differences between isolates from the same and different elephants are indicated in grey.

Genome-wide sequence data for eight isolates cultured from different organs of the three elephants were obtained. The sequencing data has been deposited in the European Nucleotide Archive (EMBL-EBI) under the study ID PRJEB21800 (see Supplementary Table S1). The read depth ranged from 19X to 160X and more than 99% of the MTBC reference genome was covered. The regions not covered primarily included members of the GC-rich and repetitive PE/PPE gene families^21^. Sequence analysis revealed that all eight isolates were human-adapted strains belonging to MTBC Lineage 4^22^ and sublineage L4.10 also referred to as PGG3^7^. When considering fixed nucleotide differences, the median pairwise distance between any two isolates was 6 SNPs, with a minimum of 0 SNPs and a maximum of 19 SNPs. The widest genetic distance (19 SNPs) was detected between the isolate originating from the gastric mucosa of Elephant 3 and the isolates from Elephant 1’s lungs (Fig. 2). However, the isolate from the pharyngeal swab of Elephant 1 and the isolate from the right lung of Elephant 3 had no fixed SNPs difference.

No phenotypic resistance was detected for the five first-line antimycobacterial antibiotics tested (isoniazid, rifampicin, ethambutol, pyrazinamide and streptomycin). WGS analysis confirmed the susceptibility of the isolates, as none of the sequences contained any drug resistance-conferring mutations, according to Walker and co-workers^23^.

## Discussion

In contrast to the trunk wash results, necropsy and bacteriological culture confirmed shedding of *M. tuberculosis* based on the positive culture results from the trunk mucosa, tracheobronchial and pharyngeal swab in two out of three elephants. Accurate and robust ante mortem diagnostic tests for elephants are missing and have been limited by challenges associated with sample collection, performance and interpretation. The DPP® VetTB Assay has been accredited for diagnostic purposes by the U.S. Department of Agriculture who assure high confidence of results^18^. Although the antigens present in this test are reported to be highly specific for the MTBC, they were also reported to be expressed by Nontuberculous Mycobacteria (NTM) species such as *M. kansasii* and *M. marinum* ^24,25^. Similarly, a recent report showed that African elephants infected with *M. szulgai* cross-reacted with antigen MPB83, leading to false-positive results and to erroneous diagnosis with potentially fatal outcome^26^. The current gold standard for ante mortem diagnosis of TB infection in elephants is mycobacterial culture from respiratory secretions obtained by trunk wash. However, due to the intermittent shedding of the pathogen, a negative culture does not rule out an infection. Moreover, elephants use their trunks for a variety of activities and culture overgrowth with fungi or NTMs is a common problem^27^. Another limitation of the current diagnostic gold standard is the relatively slow reporting time, typically 6–8 weeks necessary for culture of MTBC members. While culture results are pending, a potential infected elephant may transmit the infection to other animals or humans that come in close contact. Before the outbreak period, seven additional elephants were present at the zoo with direct contact to the three TB infected animals. Once infection with TB was suspected based on DPP® VetTB Assay results, the remaining herd was separated and kept without direct contact to the three TB infected elephants These elephants were tested at regular intervals by trunk wash sampling without detection of MTBC, and at time of writing, all were alive and in good clinical condition. Although detection of TB was only possible post mortem, the three elephants presented various additional clinical problems, mostly involving the reproductive system and the locomotor activity. Therefore, euthanasia was based on the deteriorated general condition and not uniquely on a suspected TB infection. More controversial is the management of DPP® VetTB Assay positive and trunk-wash negative animals that live in close contact with other animals and do not present any clinical signs.

The higher sensitivity of spoligotyping compared with commercial available methods, e.g. GenoType® MTBC kit (HainLifescience, Nehren, Germany), allowed species determination of the isolates directly from the tissue’s DNA extract. This enables rapid implementation of epidemiological and therapeutic measures and, at the same time, characterization of the MTBC pathogen at the species level. Directly analyzed lung tissue from each elephant revealed three identical spoligotyping profiles, suggesting a single source of infection. However, since the discriminatory power of spoligotyping is too low to infer transmission chains reliably, MLVA offers usually a better alternative^28,29^. In human TB, double-locus variations observed between different clinical isolates of *M. tuberculosis* usually reflect infection with distinct clones with no direct epidemiological linkage and therefore belonging to different transmission chains^30^. However, little is known about the genetic variability of *M. tuberculosis* within elephants. One possibility is that prolonged infection occurred. This allowed the establishment of different clonal mycobacterial herds showing a marked within-host genetic divergence. The present results indicate therefore the importance of multiple sampling for epidemiological investigation of TB in elephants (Fig. 2). The eight isolates of the present study were classified as *M. tuberculosis* Lineage 4, belonging to the so-called principal genetic group 3 (PGG3)^31^, which is geographically one of the most widespread causes of human TB worldwide. Very low genetic distance among the eight isolates was observed overall. The average distance was 6 SNPs, which is below the threshold of 12 SNPs proposed by Walker *et al*. for epidemiologically linked/unlinked cases^32^. In detail, according to the proposed thresholds, an epidemiological linkage is expected between isolates differing by five or fewer SNPs, whereas isolates that differ by more than 12 SNPs are supposed to be part of different transmission chains^32^. The elephant isolates are most likely the result of a transmission chain, potentially part of a larger cluster with human origin. Based on WGS data and MLVA genotyping, Elephant 3 showed the largest within-host genetic divergence compared with the other two animals, displaying both MLVA profiles in the right lung and a distance of 15 SNPs between the trunk and gastric mucosa isolates compared with the right lung isolate (Fig. 2 and Table 2). Furthermore, the marked divergence of 8 SNPs observed between the isolates from Elephant 1 and Elephant 2 suggests transmission events involving single distinct genotypes originating from Elephant 3, which most likely represent the spreader case. It is intriguing that two of the four isolates from Elephant 3, namely those generated from the gastric– and the trunk mucosa respectively, showed a SNPs distance above the threshold of 12 SNPs if compared with the isolates of Elephant 1 (Fig. 2). This indicates that the analysis of a single sample from Elephant 3, e.g. by trunk wash, would have led to a questionable epidemiological elucidation of the outbreak. Moreover the observed mycobacterial genetic variation within Elephant 3 suggest a particularly prolonged infection, leading to a level of clonal divergence exceeding the proposed maximal limit of single-locus variation seen in human infection and human-to-human transmission. The described results show the high discriminatory power of MLVA between genetically close *M. tuberculosis* isolates and correlate with the WGS analysis findings. Taking into account the accessibility of MLVA for laboratory with restricted resources and the straightforward performance compared to WGS analysis, MLVA represent a valid and effective technique for genotyping MTBC isolates of animal origin. An exhaustive attempt to delineate the occurred transmission between the three animals however was only possible though WGS analysis, proving a greater resolution compared to MLVA^33^.

Antituberculosis therapy among captive elephants is not standardized but sporadic successful treatments have been reported^34^. General condition of diseased animals and management of potentially MTBC shedding patients represent however crucial aspects of antimycobacterial therapy in elephants. All elephant isolates were susceptible to the antimycobacterial agents tested. Recently, novel mutations associated with streptomycin resistance have been reported in humans^35^. Although all eight genomes showed a non-synonymous mutation in gidB_Ala205Thr, this mutation has also been reported in two susceptible isolates from Belarus^35^.

Our findings confirm transmission of *M. tuberculosis* between the three elephants, and from the public health point of view, these animals constituted a potential source for TB infection in humans. On the other hand, since the *M. tuberculosis* lineage detected is very common in humans, reverse zoonotic transmission from human to elephant most likely occurred, highlighting the importance of protection of this endangered animal species from infection.

Therefore, closer collaboration between public health, veterinary and occupational health officers is recommended.

## Material and Methods

### Culture and identification of mycobacteria

Trunk wash samples were collected according to the guidelines for the diagnosis and control of TB in captive elephants with slight modifications^36^. Briefly, 250 ml of sterile saline solution were instilled into one elephant’s nostril, the trunk was lifted in order to distribute the fluid and finally the elephant exhaled the saline in a sterile collection plastic bag. Trunk wash samples were collected for each elephant in a series of three separate days within a one-week period. The collected fluid was centrifuged 15 min at 3000 × *g* and the sediment decontaminated as previously described^37^. For the microbiological analysis, two different lung localizations (left and right lung), several lymph nodes (pooled), gastric mucosa, trunk mucosa, pharyngeal- and tracheobronchial swabs were collected from each elephant and processed independently according to Ghielmetti and co-workers^37^. Decontaminated speciments, including trunk wash fluid, organ samples and swabs were tested using *artus® M. tuberculosis* PCR Kit with a Rotor-Gene Q device according to the manufacturer’s protocol (QIAGEN, Hilden, Germany) and used as inoculum for culture. Cultures showing growth of suspicious mycobacterial colonies were checked for acid-fast bacilli after Ziehl-Neelsen staining and identified as MTBC by *artus® M. tuberculosis* PCR. Suspicious mycobacterial colonies yielding negative results by MTBC specific PCR were identified by sequencing the 16S rDNA gene^38^. Subcultures were established on Middlebrook 7H10 agar plates (Becton, Dickinson & Company, Allschwil, Switzerland) using mycobacteria harvested from 1.5 ml MGIT cultures by centrifugation for 10 min at 13000 x *g*. To avoid loss of genetic variability among mycobacteria obtained by culture, multiple colonies (5 to 10) from Middlebrook 7H10 agar plates (BD) were picked for DNA extraction. Automated DNA purification was completed on a QIAcube instrument in accordance with the QIAamp cador Pathogen Mini Kit protocol (QIAGEN).

### Spoligotyping and MLVA

Spoligotyping was performed by using a commercial microarray system with integrated data analysis (Alere Technologies, Jena, Germany)^37^. For each elephant, one lung sample was tested directly by spoligotyping prior to culturing. Additionally, all isolates obtained by culture were analyzed individually. The spoligotype patterns were assigned according to the international nomenclature (SITVIT database). MLVA was performed using the standard 24 markers panel used for *M. tuberculosis* typing according to Supply *et al*.^30^. In addition, two hypervariable loci (VNTR2163a and VNTR3232) were included. The single markers were amplified by end-point PCR and subsequently analyzed using a capillary electrophoresis device^37^.

### Whole-genome sequencing and sequence analysis

Whole-genome sequencing (WGS) was performed using the Nextera XT library preparation kit and the HiSeq 2500 Illumina sequencer. The FastQ files containing the raw paired-end reads were processed using a python pipeline developed in house as follows. The reads were first adapter- and quality- trimmed with Trimmomatic v0.33^39^. Reads lower than 20 bp were not kept for the downstream analysis. Overlapping paired-end reads were then merged with SeqPrep (https://github.com/jstjohn/SeqPrep). The resulting filtered reads were mapped to a hypothetical reconstructed MTBC ancestor^22^ with BWA v0.7.12^40^. Duplicated reads were marked by the MarkDuplicates module of Picard v 2.1.1 (https://github.com/broadinstitute/picard). The RealignerTargetCreator and IndelRealigner modules of GATK v.3.4.0^41^ were used to perform local realignment of reads around indels. SNPs were called with Samtools v1.2^42^ and VarScan v2.4.1^43^ using the following thresholds: minimum mapping quality of 20, minimum base quality at a position of 20 and minimum read depth at a position of 7X. SNPs were considered fixed at a frequency of ≥90% and alleles were considered ancestral when the SNP frequency was ≤10%. Furthermore, SNPs were called only if the alternative basecall was supported by at least five reads and without strand bias. All variants were annotated using snpEff v4.11^44^, in accordance with the *M. tuberculosis* H37Rv reference annotation (AL123456.3). SNPs falling in regions with at least 50 bp identity to other regions in the genome were excluded from the analysis^45^.

The variable SNPs alignment was obtained by concatenating the SNP calls present in the variant calling file of each genome, using the IUPAC nucleotide ambiguity codes for heterozygous calls. A position was considered variable if at least one genome had a SNP at that position. Called deletions and positions not called according to the minimum threshold of 7 were encoded as gaps. Positions for which the proportion of gaps exceeded 50% were excluded from the alignment. The variable SNPs alignment was used to infer a maximum likelihood phylogeny using RaxML 8.2.8^46^, applying the general time-reversible (GTR) model of evolution. The confidence of nodes was assessed by bootstrapping 1000 pseudoreplicates, and *M. canettii* was used as an outgroup and to root the phylogeny. Number of SNPs difference between every pairwise comparison was obtained with Mega6^47^, reporting only fixed SNPs within a genome.

### Drug susceptibility testing (DST)

The World Health Organization and the U.S. Centre for Disease Control and Prevention recommend the use of liquid culture systems for DST and for improving time to detection^48–50^. To provide an effective prophylactic drug regimen for contact animals that live close to the positive patients, phenotypic antimycobacterial susceptibility testing was performed. For each elephant, one cultured isolate was tested for drug resistance using the Bactec MGIT 960 SIRE (streptomycin STR, isoniazid INH, rifampicin RMP and ethambutol EMB) Kits and the 960 PZA (pyrazinamide PZA) Kit according to manufacturer’s instructions (BD).

### Data Availability

The datasets generated and analysed during the current study are available in the European Nucleotide Archive (EMBL-EBI) under the study ID PRJEB21800. http://www.ebi.ac.uk/ena/data/view/PRJEB21800.

## Electronic supplementary material

Supplementary Information
