# Supplementary material for: Tuberculosis in Swiss captive Asian elephants: microevolution of Mycobacterium tuberculosis characterized by multilocus variable-number tandem-repeat analysis and whole-genome sequencing
Source: Sci Rep. 2017 Nov 7;7:14647. doi: 10.1038/s41598-017-15278-9 (PMC5676744; doi:10.1038/s41598-017-15278-9)
Supplement: Supplementary file 1 — Supplementary Information [file 41598_2017_15278_MOESM1_ESM.pdf]

Tuberculosis in Swiss captive Asian elephants: microevolution of *Mycobacterium tuberculosis* characterized by multilocus variable-number tandem-repeat analysis and whole-genome sequencing

Giovanni Ghielmetti, Mireia Coscolla, Maja Ruetten, Ute Friedel, Chloé Loiseau, Julia Feldmann, Hanspeter W. Steinmetz, David Stucki, Sebastien Gagneux

Sample names with corresponding Sample accession and Run accession IDs

Sample name	Sample accession	Run accession
G08157	SAMEA104164785	ERR2036897; ERR2036898; ERR2036899
G08158	SAMEA104164786	ERR2036900; ERR2036901; ERR2036902
G08159	SAMEA104164787	ERR2036903; ERR2036904; ERR2036905
G08160	SAMEA104164788	ERR2036906; ERR2036907; ERR2036908
G08161	SAMEA104164789	ERR2036909; ERR2036910; ERR2036911
G08162	SAMEA104164790	ERR2036912; ERR2036913; ERR2036914
G08163	SAMEA104164791	ERR2036915; ERR2036916; ERR2036917
G08164	SAMEA104164792	ERR2036918; ERR2036919; ERR2036920
